# Adenocarcinoma Developing Overlying a Colonic Lipoma: A Case Report

**DOI:** 10.70352/scrj.cr.26-0198

**Published:** 2026-08-29

**Authors:** Yasuma Nakata, Kazunosuke Yamada, Akiko Ichihara, Roko Hamada, Daisuke Hara, Takumi Kiwaki, Yoshiko Umekita, Yuichiro Sato, Shinsuke Takeno

**Affiliations:** 1 Department of Surgery, Faculty of Medicine, University of Miyazaki, Miyazaki, Miyazaki, Japan; 2 Department of Pathology, Section of Oncopathology and Morphological Pathology, Faculty of Medicine, University of Miyazaki, Miyazaki, Miyazaki, Japan

**Keywords:** colonic lipoma, colorectal cancer, adenocarcinoma, submucosal tumor, case report

## Abstract

**INTRODUCTION:**

Colonic lipomas are benign mesenchymal tumors composed of mature adipocytes and are most commonly located in the submucosal layer. They are usually asymptomatic and are often managed conservatively. Although adenomas arising overlying colonic lipomas have been reported, the coexistence of adenocarcinoma directly overlying a lipoma is extremely rare. The pathogenic relationship between submucosal tumors and epithelial malignancies remains unclear.

**CASE PRESENTATION:**

A 76-year-old man was diagnosed with a lipoma in the descending colon (diameter, approximately 30 mm) and was managed with surveillance. After 26 months of follow-up, colonoscopy revealed a newly developed small type 2 ulcerative lesion located directly over the lipoma. A biopsy confirmed adenocarcinoma. Endoscopic ultrasonography demonstrated a homogeneous hyperechoic lesion in the 3rd layer consistent with a lipoma, along with focal thickening of the 2nd layer, without evidence of deep invasion. CT and MRI showed imaging features characteristic of a lipoma, with a peripheral component suspicious for malignancy. The patient was diagnosed with descending colon cancer (cT2N0M0, Stage I) and underwent laparoscopic left hemicolectomy with lymph node dissection. A histopathological examination revealed a lipoma extending from the submucosa into the muscularis propria, while the carcinoma was confined to the mucosa and muscularis propria overlying the lipoma, without invasion into the lipomatous tissue. No histological findings indicative of chronic inflammation were observed.

**CONCLUSIONS:**

We report a rare case of adenocarcinoma developing directly over a colonic lipoma during surveillance. Although chronic inflammation has been proposed as a possible mechanism linking submucosal tumors to epithelial malignancies, no such features were identified in this case, and a causal relationship could not be established. While colonic lipomas are generally managed conservatively, careful endoscopic surveillance may be considered in selected patients with relatively large lipomas requiring long-term follow-up.

## Abbreviations

ESCC
: esophageal squamous cell carcinoma
EUS
: endoscopic ultrasonography
TNM
: tumor–node–metastasis
UICC
: Union for International Cancer Control

## INTRODUCTION

Lipoma is a benign soft tissue tumor composed of mature adipocytes that was first described by Bauer in 1757.^1)^ Although lipomas most commonly arise in subcutaneous tissues, they may also develop in deeper anatomical locations. Most lipomas are soft, highly mobile, and typically asymptomatic.

Colorectal cancer is one of the most common malignancies worldwide. Chronic inflammation and inflammation-driven immune responses play a critical role in colorectal carcinogenesis by promoting genetic alterations and the progression from normal epithelium to adenoma and carcinoma.^2)^

Although several cases of adenoma arising overlying colonic lipomas have been reported^3–5)^ (**Table 1**), cases with coexisting adenocarcinoma are extremely rare. We report a case of adenocarcinoma located directly over a lipoma in the descending colon, along with a review of the relevant literature.

**Table 1:** Reported cases of colonic lipomas with overlying mucosal lesions

Ref.	Sex/age	Location	Size of lipoma	Type of mucosal lesion	Size of mucosal lesion
Yeom et al. (2013)^3)^	F/68	Ascending colon	9 mm	Hyperplastic and serrated epithelium	9 mm
Radhi et al. (1997)^3)^	M/45	Sigmoid colon	Not mentioned	Hyperplastic and serrated epithelium	Not mentioned
Chu et al. (2009)^3)^	F/52	Ascending colon	30 mm	Tubular adenoma	20 mm
Nguyen et al. (2021)^3)^	M/71	Hepatic flexure	Not mentioned	Tubular adenoma	20 mm
Moschetta et al. (2018)^3)^	M/49	Transverse colon	30 mm	Tubulovillous adenoma	Not mentioned
Osera et al. (2020)^4)^	M/79	Transverse colon	Not mentioned	Tubular adenoma	12 mm
Oura et al. (2023)^5)^	M/80	Transverse colon	20 mm	Tubular adenoma	10 mm

F, female; M, male

## CASE PRESENTATION

**Fig. 1:**
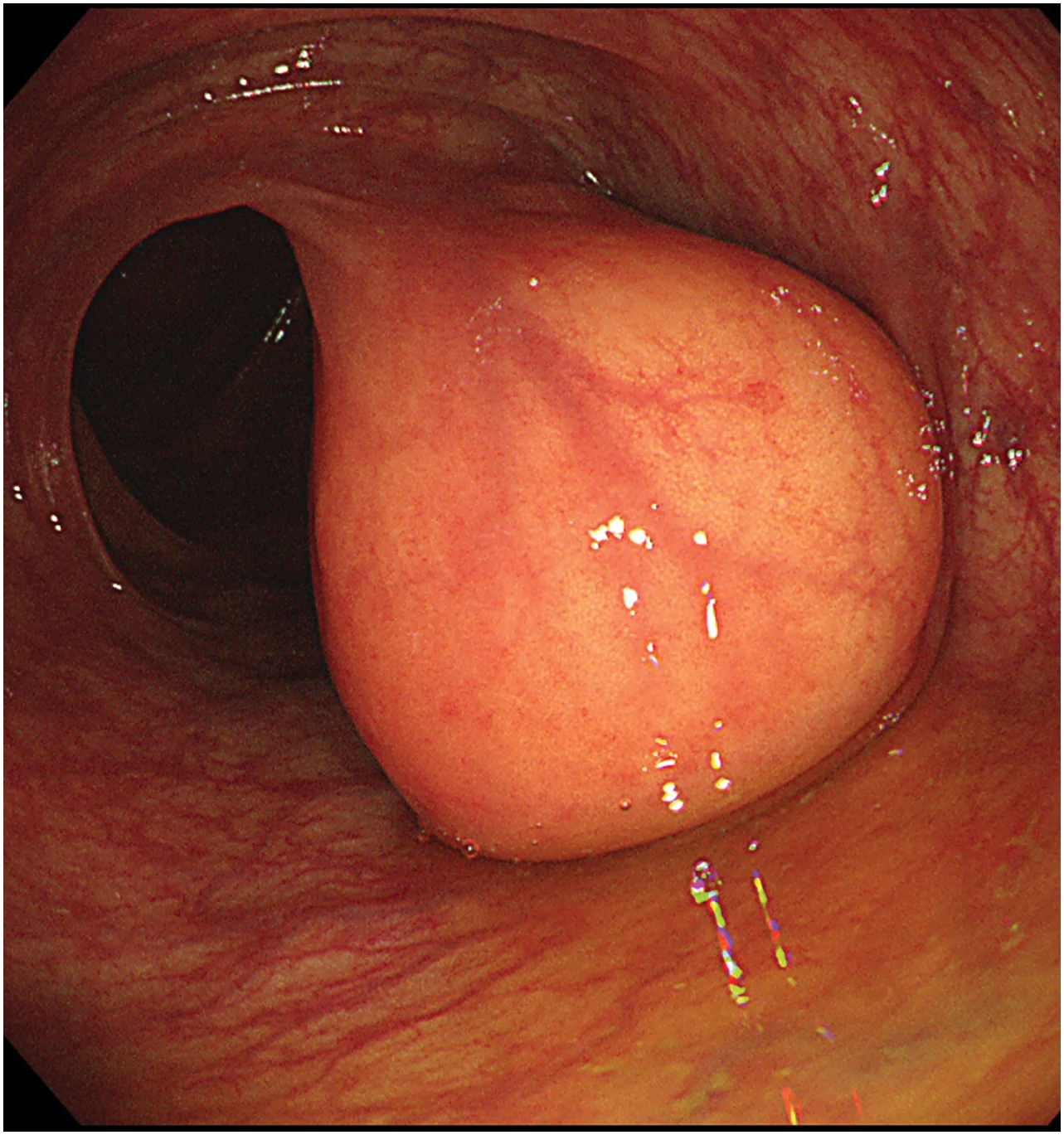
Initial colonoscopy. No neoplastic lesion was observed on the initial colonoscopic examination.

**Fig. 2:**
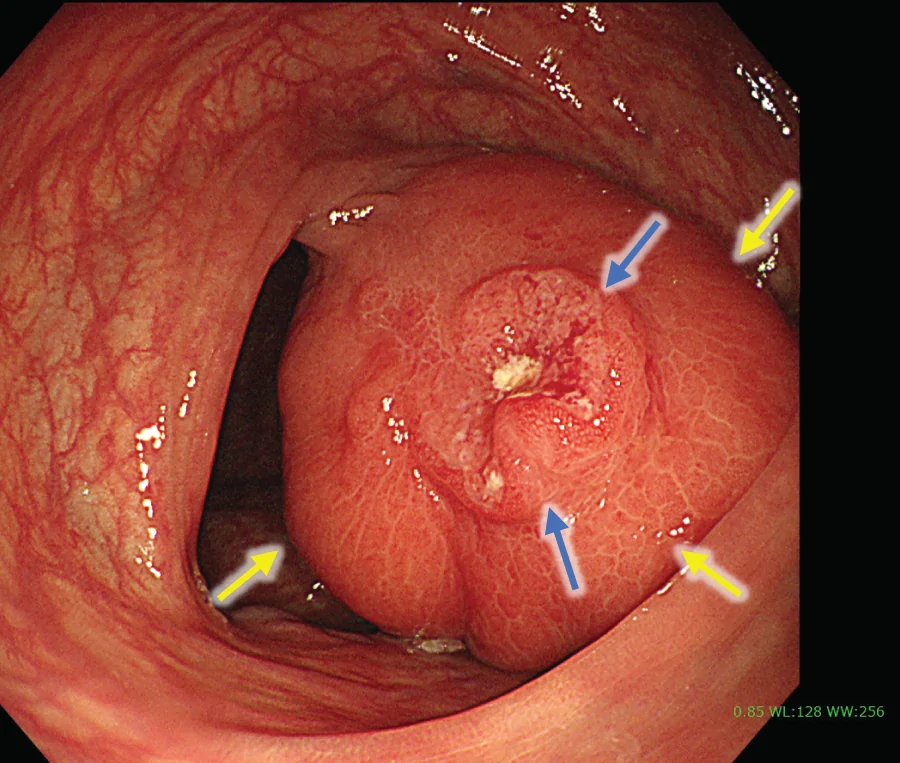
Colonoscopic findings of the colonic tumor. Blue arrows: Type 2 tumor. Yellow arrows: Lipoma. A type 2 tumor is observed directly overlying the lipoma.

**Fig. 3:**
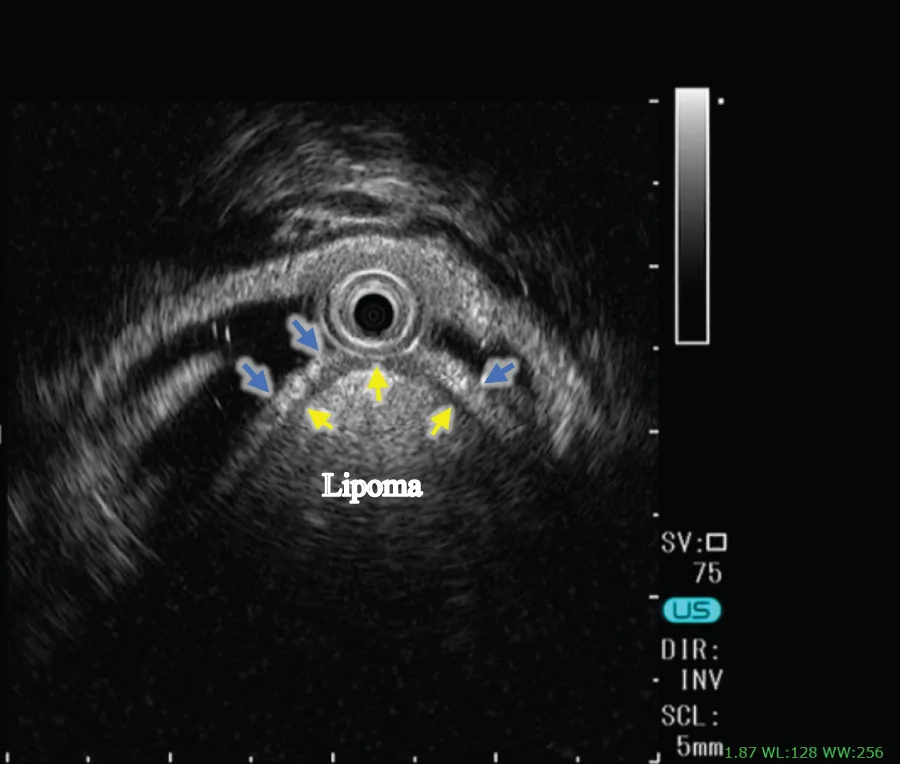
EUS findings of the tumor and lipoma. Blue arrows: Superficial mucosa (hyperechoic area). Yellow arrows: Deep mucosa (hypoechoic area). Lipoma: A homogeneous hyperechoic area was observed in the 3rd layer, which was consistent with a lipoma. EUS, endoscopic ultrasonography

**Fig. 4:**
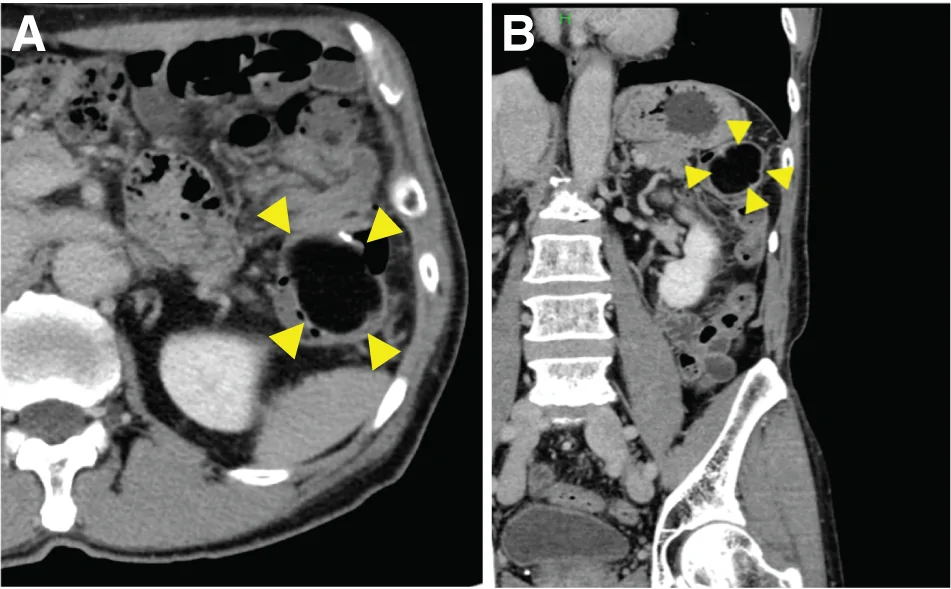
Contrast-enhanced CT findings. A 35 × 30-mm lipoma was identified on contrast-enhanced CT (yellow arrowhead). (**A**) Axial view; (**B**) coronal view.

The patient was a 76-year-old man who had been diagnosed with a lipoma in the descending colon 2 years and 2 months prior based on colonoscopy (**Fig. 1**). As the lesion measured approximately 30 mm in diameter and was asymptomatic, the patient was managed with observation, including annual CT and biennial colonoscopy. During follow-up colonoscopy, a newly developed small type 2 tumor was identified directly overlying the lipoma (**Fig. 2**). A biopsy was performed, and the diagnosis was adenocarcinoma. The patient was referred to our hospital for further evaluation and treatment. Colonoscopy revealed a 20-mm submucosal tumor in the splenic flexure of the descending colon, with a 10-mm ulcerative lesion exhibiting an irregular margin and a depression just above it. Scope passage was possible. The area suspected to represent a type 2 tumor was firm on palpation, whereas the smooth-surfaced portion showed a positive cushion sign. EUS demonstrated a homogeneous hyperechoic area in the 3rd layer, suggestive of a lipoma. In addition, focal thickening of the 2nd layer with a localized mucosal defect at the center of the thickened mucosa was observed, strongly suggesting advanced colorectal cancer. However, the boundary between the thickened 2nd layer and the hyperechoic area of the 3rd layer remained clearly demarcated, and no obvious invasive tendency was identified (**Fig. 3**). The boundary between the 2nd and 3rd layers remained distinct, and no definitive evidence of invasive features could be identified at this level. CT showed a 36 × 29-mm low-attenuation area in the descending colon (**Fig. 4**). No evidence of descending colon cancer was identified, and there were no significant lymph node enlargements or suspicious lesions indicating liver or lung metastasis.

**Fig. 5:**
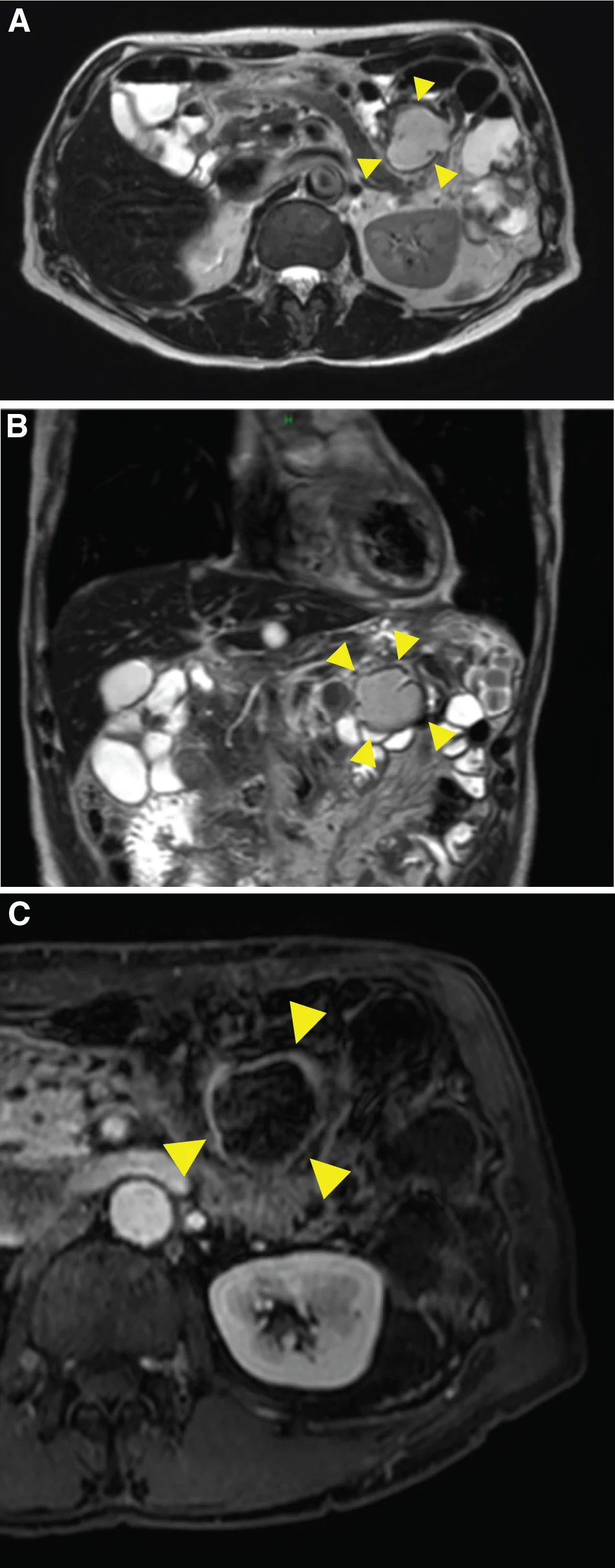
MRI findings. (**A**, **B**) A high-signal-intensity mass on T2-weighted imaging (yellow arrowheads). (**C**) A low signal intensity on fat-suppressed T2-weighted imaging with contrast enhancement overlying the lipoma (yellow arrowheads).

**Fig. 6:**
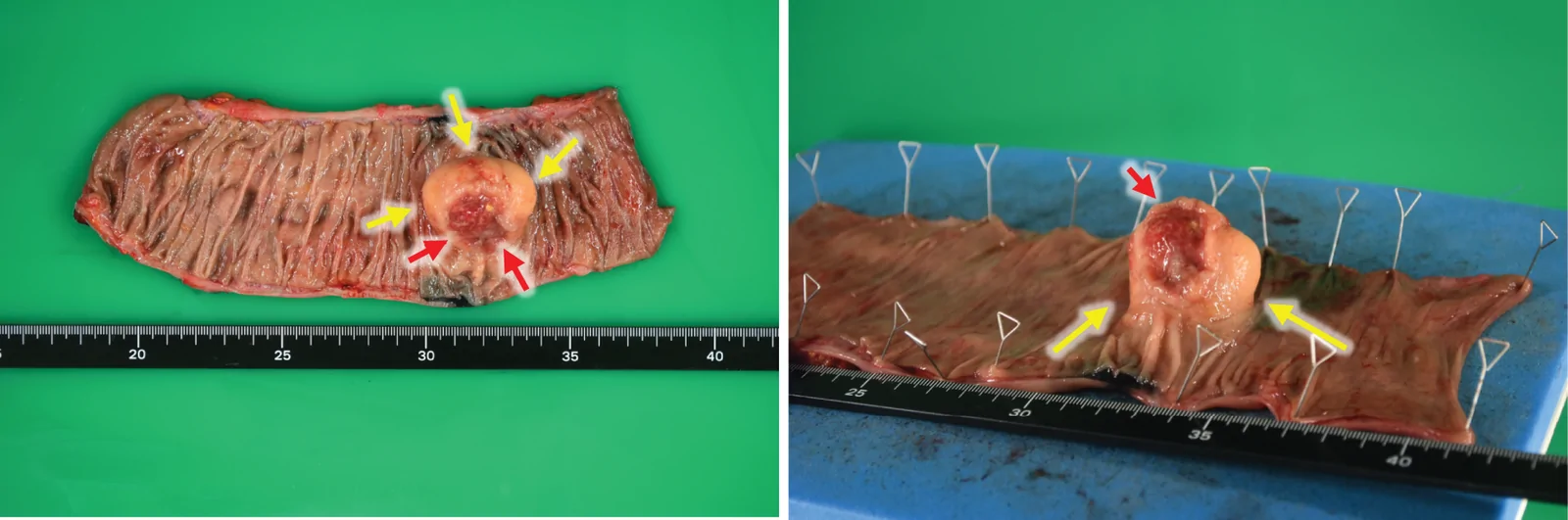
Macroscopic findings. Red arrows: Type 2 tumor. Yellow arrows: Lipoma. Consistent with the endoscopic findings, a type 2 tumor was observed directly overlying the lipoma.

MRI revealed a tumor lesion in the descending colon exhibiting high signal intensity on both T1-weighted and T2-weighted sequences. The lesion demonstrated low signal intensity on fat-suppressed T2-weighted images, consistent with a lipoma. Additionally, a hyperintense area was observed at the periphery of the lipoma on fat-suppressed T2-weighted imaging, suggestive of an associated malignant component (**Fig. 5**). The diagnosis was descending colon cancer [D, Type 2, cT2, cN0, cM0, cStage I] (stage classification was based on the TNM staging system defined by the UICC, 8th edition). The patient underwent laparoscopic left hemicolectomy with intermediate lymph node dissection, and his postoperative course was uneventful (**Fig. 6**). The patient was discharged home on POD 14.

**Fig. 7:**
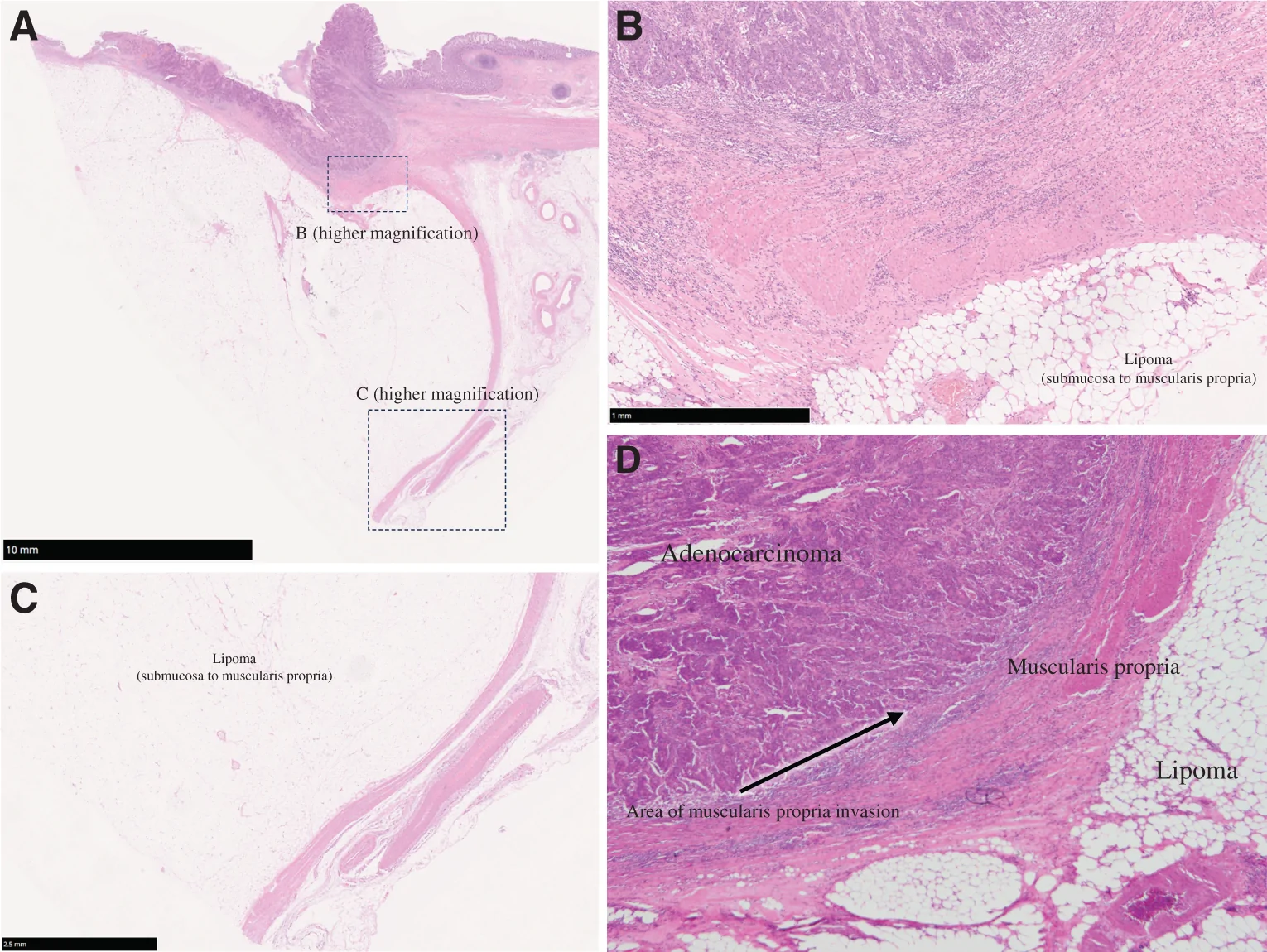
Microscopic findings. Hematoxylin and eosin-stained sections. (**A**) Low-power view showing a colonic lipoma extending from the submucosa to the muscularis propria, with adenocarcinoma present in the overlying mucosa. (**B**) Higher magnification demonstrating moderately differentiated tubular adenocarcinoma (tub2). (**C**) The carcinoma was confined to the mucosal layer overlying the lipoma, and no invasion into the lipomatous tissue was observed. (**D**) Higher magnification showing the area of muscularis propria invasion. The carcinoma invaded the muscularis propria but did not directly infiltrate the underlying lipomatous tissue.

Histopathological examination revealed a 40 × 35-mm lipoma extending from the submucosal layer into the muscularis propria. The carcinoma was confined to the superficial layer overlying the lipoma, and a thinned muscularis propria was identified beneath the lipoma (**Fig. 7**). These findings indicated that the carcinoma developed independently overlying the lipoma rather than arising from or invading into the lipomatous tissue.

The final pathological diagnosis was pT2, pN0, cM0, Stage I. No histological features suggestive of chronic inflammation, including mononuclear cell infiltration, tissue destruction, angiogenesis, or fibrosis, were observed.

## DISCUSSION

Concerning gastrointestinal tract lipomas, they are classified into submucosal, subserosal, intramuscular, and mixed types; approximately 90% develop in the submucosa and 10% in the subserosal layer.^6)^ The incidence of colonic lipoma ranges from 0.2% to 4.4%, accounting for approximately 1.8% of benign colonic lesions, making it the 3rd most common benign tumor after hyperplastic and adenomatous polyps.^6)^

Previous studies have demonstrated that colonic lipomas predominantly occur in women of 50–60 years of age, with a predilection for the right colon (cecum and ascending colon), where 70%–90% of cases are located.^6,7)^ Clinical manifestations include abdominal pain, diarrhea, constipation, and hematochezia, and larger lesions may cause intestinal obstruction.^1)^ Notably, 25% of lipomas >20 mm become symptomatic, and 75% of those >40 mm are reported to present with symptoms.^8)^ Diagnostic modalities include abdominal ultrasonography, CT, MRI, and EUS.^6)^ Endoscopic hallmarks include the “cushion sign,” “naked fat sign,” and “tenting sign.”

Treatment options consist of endoscopic resection and surgical excision. Endoscopic resection may be considered for lesions <20 mm, whereas surgical resection should be considered for lesions >40 mm.^8)^ Jiang et al. further proposed that surgical resection is advisable for lesions >40 mm, broad-based lesions without a stalk, those with an uncertain preoperative diagnosis, or cases in which severe symptoms render endoscopic removal difficult.^9)^ However, there is currently no consensus regarding the optimal management strategy for lipomas measuring 20–40 mm. At present, no clear consensus has been established regarding the optimal management of colonic lipomas.

In this report, we describe a rare case in which a small type 2 tumor developed overlying a colonic lipoma that had been managed with surveillance. Reports of adenocarcinoma arising overlying a colonic lipoma are extremely rare. In our case, a type 2 lesion was identified overlying a descending colon lipoma after a follow-up period of 26 months, and the patient subsequently underwent laparoscopic left hemicolectomy with intermediate lymph node dissection. A histopathological examination revealed that the lipoma extended from the submucosal layer into the muscularis propria, while the carcinoma had invaded up to the surface of the lipoma.

In the upper gastrointestinal tract, subepithelial tumors have been suggested to promote the development of epithelial malignancies. Iwaya et al. reported a case of ESCC arising overlying an esophageal leiomyoma and summarized 12 cases of ESCC developing on esophageal submucosal tumors, including 9 leiomyomas and 3 lipomas.^10)^ In addition, Yamamoto et al. reported a case of poorly differentiated adenocarcinoma with a superficial spreading pattern (type IIc + IIb) arising on a gastric lipoma, characterized by widespread cancer cell distribution over the lipoma surface. Including 2 previously reported cases, only 3 similar cases have been documented.^11)^ While squamous cell carcinoma was observed in the esophagus, adenocarcinoma was identified in the stomach in the present case, suggesting carcinogenesis derived from epithelial tissue specific to each anatomical site. These reports suggest that submucosal tumors may potentially contribute to the development of epithelial malignancies; however, the underlying mechanism remains unclear and largely speculative. Chronic inflammation induced by persistent mechanical stimulation has been proposed as one possible explanation. In the present case, however, histological findings suggestive of chronic inflammation, including mononuclear cell infiltration, tissue destruction, neovascularization, or fibrosis, were absent, making this mechanism less likely.

Nevertheless, several alternative hypotheses may also be considered. Repeated mechanical irritation or mucosal trauma caused by friction between the protruding lesion and fecal contents may contribute to epithelial injury. In addition, chronic compression of the overlying mucosa by the lipoma could theoretically induce localized ischemia or increased mucosal vulnerability. Altered local bowel dynamics and fecal stasis around the elevated lesion may also result in persistent mucosal stimulation. However, these mechanisms remain hypothetical, and there is currently insufficient evidence to support a direct causal relationship between colonic lipomas and carcinogenesis.

Therefore, the coexistence of adenocarcinoma and lipoma in the present case may represent an incidental association rather than a true pathogenetic interaction. Further accumulation of similar cases is necessary to clarify whether submucosal tumors play any role in colorectal carcinogenesis.

Previous reports have also described carcinoma arising in association with colonic lipomas. Mayo et al. reviewed 164 gastrointestinal lipomas, including 119 colonic lipomas (72.5%), and reported that 48 cases (29%) were associated with a coexisting malignant neoplasm (gastric cancer, n = 1; colorectal cancer, n = 47). In at least 30 of these cases, the lipoma and carcinoma were located in close proximity, and in 4 cases, the 2 lesions were within 5 cm of each other.^12)^ However, most of these reports were based on relatively old literature, and similar cases have rarely been documented in recent years. Advances in endoscopic imaging, pathological diagnosis, and colorectal cancer screening may have influenced the apparent frequency and interpretation of such coexistence. Therefore, the relationship between colonic lipomas and colorectal carcinoma remains controversial, and whether these lesions represent incidental coexistence or a true pathogenetic association remains unclear. While the coexistence of submucosal tumors and epithelial malignancies may be incidental, a pathogenic association cannot be completely excluded. In the present case, chronic inflammation was unlikely to have contributed to carcinogenesis; however, the involvement of other carcinogenic factors cannot be ruled out. Accordingly, when a submucosal tumor is identified, careful surveillance is warranted, as malignancies may arise in the mucosa directly overlying the lesion.

The coexistence observed in this case may be incidental, and a causal relationship has not been established. However, the development of carcinoma during surveillance suggests that relatively large colonic lipomas may warrant careful long-term observation, although the optimal management strategy remains unclear.

## CONCLUSIONS

We report a rare case in which an advanced carcinoma developed directly overying a colonic lipoma during a 26-month follow-up period. Although no chronic inflammation was observed pathologically, several reports have discussed a possible association between submucosal tumors and epithelial carcinogenesis; however, the underlying mechanism remains speculative. Colonic lipomas are often managed conservatively, but this case may highlight the importance of careful surveillance. Further research is needed to clarify the optimal management strategy for colonic lipomas.
